# Reproducible supervised learning-assisted classification of spontaneous synaptic waveforms with Eventer

**DOI:** 10.3389/fninf.2024.1427642

**Published:** 2024-09-13

**Authors:** Giles Winchester, Oliver G. Steele, Samuel Liu, Andre Maia Chagas, Wajeeha Aziz, Andrew C. Penn

**Affiliations:** School of Life Sciences, University of Sussex, Brighton, United Kingdom

**Keywords:** machine learning, event detection, synapses, analysis, reproducibility

## Abstract

Detection and analysis of spontaneous synaptic events is an extremely common task in many neuroscience research labs. Various algorithms and tools have been developed over the years to improve the sensitivity of detecting synaptic events. However, the final stages of most procedures for detecting synaptic events still involve the manual selection of candidate events. This step in the analysis is laborious and requires care and attention to maintain consistency of event selection across the whole dataset. Manual selection can introduce bias and subjective selection criteria that cannot be shared with other labs in reporting methods. To address this, we have created Eventer, a standalone application for the detection of spontaneous synaptic events acquired by electrophysiology or imaging. This open-source application uses the freely available MATLAB Runtime and is deployed on Mac, Windows, and Linux systems. The principle of the Eventer application is to learn the user's “expert” strategy for classifying a set of detected event candidates from a small subset of the data and then automatically apply the same criterion to the remaining dataset. Eventer first uses a suitable model template to pull out event candidates using fast Fourier transform (FFT)-based deconvolution with a low threshold. Random forests are then created and trained to associate various features of the events with manual labeling. The stored model file can be reloaded and used to analyse large datasets with greater consistency. The availability of the source code and its user interface provide a framework with the scope to further tune the existing Random Forest implementation, or add additional, artificial intelligence classification methods. The Eventer website (https://eventerneuro.netlify.app/) includes a repository where researchers can upload and share their machine learning model files and thereby provide greater opportunities for enhancing reproducibility when analyzing datasets of spontaneous synaptic activity. In summary, Eventer, and the associated repository, could allow researchers studying synaptic transmission to increase throughput of their data analysis and address the increasing concerns of reproducibility in neuroscience research.

## 1 Introduction

Detection, and analysis of, spontaneous synaptic currents is a frequently chosen method by experimental neuroscience researchers to evaluate the properties of synapses (Biane et al., 2021; Williams and Featherstone, 2014; Vyleta and Smith, 2008). Recordings of spontaneous synaptic currents or potentials are technically simple to acquire but can be laborious to analyse. Whilst individual synaptic currents at typical central synapses are reported to be approximately 10 pA (Auger and Marty, 1997; Glasgow et al., 2019), this is likely to be an overestimate as only the larger synaptic currents are detectable above the noise and vary substantially between labs, even where the conditions of the experiments are largely consistent. Discerning synaptic currents from background noise can be a particularly challenging task and typically involves some element of subjective manual selection by an experienced researcher. The challenges in the analysis process are exemplified when the researcher is required to scrutinize hundreds, if not thousands, of events, increasing not only the duration of the analysis but also the chance for human error.

To avoid the burden of manually screening hours of recording time to identify synaptic currents, several semi-automated solutions have been developed to break the process down into two tasks: (1) automatic detection of plausible candidate synaptic currents, and (2) manually scrutinizing the detections, for example, labeling the flagged candidate events as true or false positive detections. The initial automatic step has largely been achieved using one of two main approaches: those that require a template waveform of a synaptic current and those that do not. The template approach computes some measure of “likeness” of the recording at each time point to the template waveform. The “likeness” of the template can represent how well it fits to the data as it slides from one sample point to the next along the recording in the time domain (Clements and Bekkers, 1997; Jonas et al., 1993). Alternatively, “likeness” can be derived by deconvolution and represents how well frequency components in the template waveform match those in the recording at each point in time in the recording. FFT-based deconvolution involves converting the recorded wave and a convolution kernel (i.e., a template waveform of the synaptic current) to the frequency domain, dividing them, and then transforming the result back to the time domain. The result is a detector trace that contains what resembles a series of sharp spikes, each of which indicates the time of onset of a synaptic event. Generally speaking, frequency domain deconvolution is more robust than time domain template matching in cases where synaptic currents are (partially) overlapping (Pernía-Andrade et al., 2012). The developments of these event detection methods (amongst others, e.g., Merel et al., 2016) have both helped to improve the sensitivity of detecting small but “true” candidate synaptic currents, and provide some screening of large, but otherwise implausible waveforms in the recordings.

Whilst automatic detection methods proceed much faster than manually screening the recordings, the classification of the synaptic currents based on those detection criteria alone is seldom sufficient in accuracy to convince users that manual intervention is not required. Many users, experts through years of manual analysis, do not trust automated or semi-automated approaches due to the output event selection not conforming to the opinion of the user. Although most commonly used software packages implement one or more of the above automatic detection methods, they also enable users to follow this up by editing the detection and/or classification of flagged candidates. However, expert manual classification of candidate synaptic currents introduces other issues, particularly with respect to the ability to reproduce how recordings are analyzed and the results obtained from them. Furthermore, many of the automatic detection methods depend heavily on setting a detection threshold (Ankri et al., 1994; Maier et al., 2011), the decision of which is effectively a compromise between true positive and false positive detection rates and therefore also influenced by the workload anticipated by the user to screen candidates during manual classification. A solution to these problems is to harness recent developments in rapid machine learning methods to learn and emulate our classification strategy. Not only does this have the potential to save users time and effort by dispensing with most (if not all) manual screening tasks, but it also opens the possibility of using common or shared models for event classification. Since computers are not nearly concerned with how many candidates need classifying, we can also compromise less on the choice of the detection threshold by setting the threshold lower.

An the task of classification can be automated using computers via an approach known as machine learning. Machine learning aims to fit models to data using statistical algorithms. There are two broad categories of machine learning, and then a multitude of classifications thereafter, which are reviewed extensively elsewhere (Greener et al., 2022). At the most basic level, machine learning can be supervised, requiring user-labeled training data, or unsupervised, where the model fitting is done independent of user-labeled training data. The advent and development of machine learning offer a potential solution to increase the accuracy and reliability of synaptic event detection whilst also decreasing the time required to perform this step. The use of machine learning in synaptic event detection is expected to remove both human errors and avoid the possibility of unconscious human bias when analyzing data from different experimental conditions. Indeed, synaptic event detection software utilizing machine learning has emerged recently (Zhang et al., 2021; Pircher et al., 2022). However, several forms of machine learning can require extensive training sets and be exceptionally computationally demanding, limiting their applicability in a basic research environment. Furthermore, the existing tools are not distributed with a graphical user interface to facilitate users to engage with the process of training a machine learning model.

The problem of manually screening candidate synaptic events is essentially a classification problem, which humans solve presumably by considering many visual features in the waveform (e.g., shape, scale, etc.). Random forest classification algorithms are particularly well suited to such binary classification problems, where a set of (largely uncorrelated) features of the events can be readily defined and measured. Briefly, this algorithm consists of generating multiple decision trees with variations between them. Whereas a single decision tree tends to be overfitted to the training dataset and thus change dramatically on a new dataset, the ensemble classification from multiple decision trees in a random forest is less prone to overfitting. The problem of overfitting is partly overcome by generating each decision tree from a replica of the data generated by random sampling with replacement (i.e., bootstrap resampling) from the initial data. Additionally, only a random subset of the predictors, alternatively called features, for the final classification is applied within each decision tree. Not only does this further overcome overfitting bias, but it also aids in lessening the impact of predictor over-emphasis. In the specific example of synaptic event detection, the dataset would be the pool of possible events, and the predictors could be amplitude, decay, halfwidth, etc. Thus, when applied to synaptic event detection, random forest classification can offer more robust ways of classifying events. Random forest classification algorithms are just that—classification algorithms, not detection algorithms, and therefore they require candidate events to first be detected. One particularly robust detection methodology is a deconvolution-based methodology first proposed by Pernía-Andrade et al. (2012). In this approach, FFT-based deconvolution is achieved in the frequency domain, whereby the resultant deconvolution wave represents how well the frequency components of the recorded signal match the event template at each point in time. A threshold is then set on this deconvolution wave, and events are proposed if the peak of the deconvolution wave passes this threshold. FFT is an implementation of the discrete Fourier transform (DFT) in which the relationship between the time domain and the frequency domain is revealed; however, it is much more computationally efficient.

In this article, we show that, following FFT-based deconvolution, a simple machine learning paradigm can effectively be applied to the problem of classifying synaptic events during the analysis of spontaneous synaptic currents. Specifically, FFT-based deconvolution is used initially to identify candidate events before either training a random forest-based machine learning model or applying a previously trained model to the classification problem on new data. Importantly, the users have the option to control the generation of the model to reproduce their classification in a reproducible way. The software was developed in MATLAB and is accompanied with a cross-platform graphical user interface (GUI) that is intuitive to use and open source. In terms of software development, Eventer provides a framework with opportunities for tuning the existing Random Forest implementation (e.g., feature set) or even adding different types of machine learning for classification (Zhang et al., 2021; Pircher et al., 2022; Wang et al., 2024; O'Neill et al., 2024). In Section 3, Eventer is shown to be able to accurately reproduce manual detection of synaptic currents and do so in only a fraction of the time. Furthermore, the use of a single conserved model trained in Eventer can increase the consistency of analysis between users. As such, the online model repository and website created to enable users around the world to deposit models of their own and use models of others are highlighted. This article outlines the user workflow for Eventer, provides a basic description of how it works, evaluates its ability to overcome some of the issues around consistency and reproducibility of analysis, and discusses various machine learning approaches that have been described recently for the purposes of synaptic event detection.

### 1.1 Eventer workflows: GUI

#### 1.1.1 Input

The GUI for the Eventer synaptic event detection analysis software was written in MATLAB's “Appdesigner.” MATLAB-based GUI can be compiled for most commonly used operating systems, thus making Eventer cross-platform. In addition to being cross-platform, Eventer also supports a wide range of the most commonly used formats in electrophysiological experiments (Table 1). Multiple file format support is largely provided by the “*ephysIO.m*” code written by Penn A. (2024).

**Table 1:** The supported file formats for data input, output and figures within Eventer.

**Data**		**Figures**
**Input format**	**Output format**	**Output format**
Binary	Binary	Raster
pCLAMP Axon binary files 1 and 2 (.abf)	Axon binary files v1.83 (^*^.abf, integer-type)	PNG (24-bit, ^*^.png)
Axograph binary files (^*^.axgx, ^*^.axgd)	Neurodata without borders v2.6.0 (^*^.nwb)	BMP (24-bit) (^*^.bmp)
HEKA PatchMaster/Pulse/ChartMaster binary files (^*^.dat)	Stimfit binary (HDF5) files (^*^.h5)	TIFF (24-bit, not compressed) (^*^.tif)
CED Spike2 binary files (^*^.smr)	ephysIO HDF5 (Matlab v7.3) binary files (^*^.phy)	TIFF (24-bit, LZW compressed) (^*^.tif)
CED Signal binary files (^*^.cfs) (*windows only*)	Text	Vector
LabVIEW Signal Express TDMS binary files (^*^.tdms)	Igor text files (^*^.itx)	EMF Windows metafile (^*^.emf)
WinEDR binary files (^*^.EDR)	Axon text files (^*^.atf)	EPS Encapsulated postscript level 3 (color) (^*^.eps)
WinWCP binary files (^*^.wcp)	ASCII comma-separated values (^*^.csv) (waves in columns)	SVG Scalable Vector Graphics (^*^.svg)
Igor Packed experiment binary files (^*^.pxp)	ASCII tab-delimited text (^*^.txt) (waves in columns)	Other
Igor binary wave files (^*^.ibw, ^*^.bwav) (v2 and v5 only)	ASCII tab-delimited text (^*^.asc) (waves stacked)	MATLAB figure (^*^.fig)
ACQ4 binary (HDF5) files (^*^.ma) (no compression only)		
WaveSurfer binary (HDF5) files (^*^.h5)		
PackIO binary files (^*^.paq)		
GINJ2 MATLAB binary files (^*^.mat)		
Neurodata without borders binary (HDF5) file v2 (^*^.nwb)		
Eventer analysis file (.evt)		
ephysIO HDF5/MATLAB binary files (^*^.phy)		
Generic 16-bit integer raw binary files (^*^.dat, ^*^.bin)		
Text		
Igor text files (^*^.itx,^*^.awav)		
Axon text files (^*^.atf)		
ASCII tab-delimited text files (^*^.txt) (± header)		
ASCII comma-separated values text files (^*^.csv) (± header)		

Functionality here for data file formats is largely provided by the ephysIO.m code written by Dr. Andrew Penn. At the time of writing, support for additional binary data formats is enabled through the use of third-party code listed here, and packaged with ephysIO: readMeta.m from ACQ4 (Luke Campagnola), IBWread.m from Jakub Bialek (modified by AP), import_wcp.m from David Jaeckeld (modified by AP), importaxo.m from Marco Russo, loadDataFile.m from WaveSurfer, ImportHEKA.m from Malcolm Lidierth and Sammy Katta (modified by AP), abfload.m from Harald Hentschke, Forrest Collman, and Ulrich Egert (modified by AP), matcfs32 and matcfs64d from Jim Colebatch, SON2 from Malcolm Lidierth, TDMS Reader from Jim Hokanson, matnwb from Lawrence Niu and Ben Dichter.

**Figure 1:**
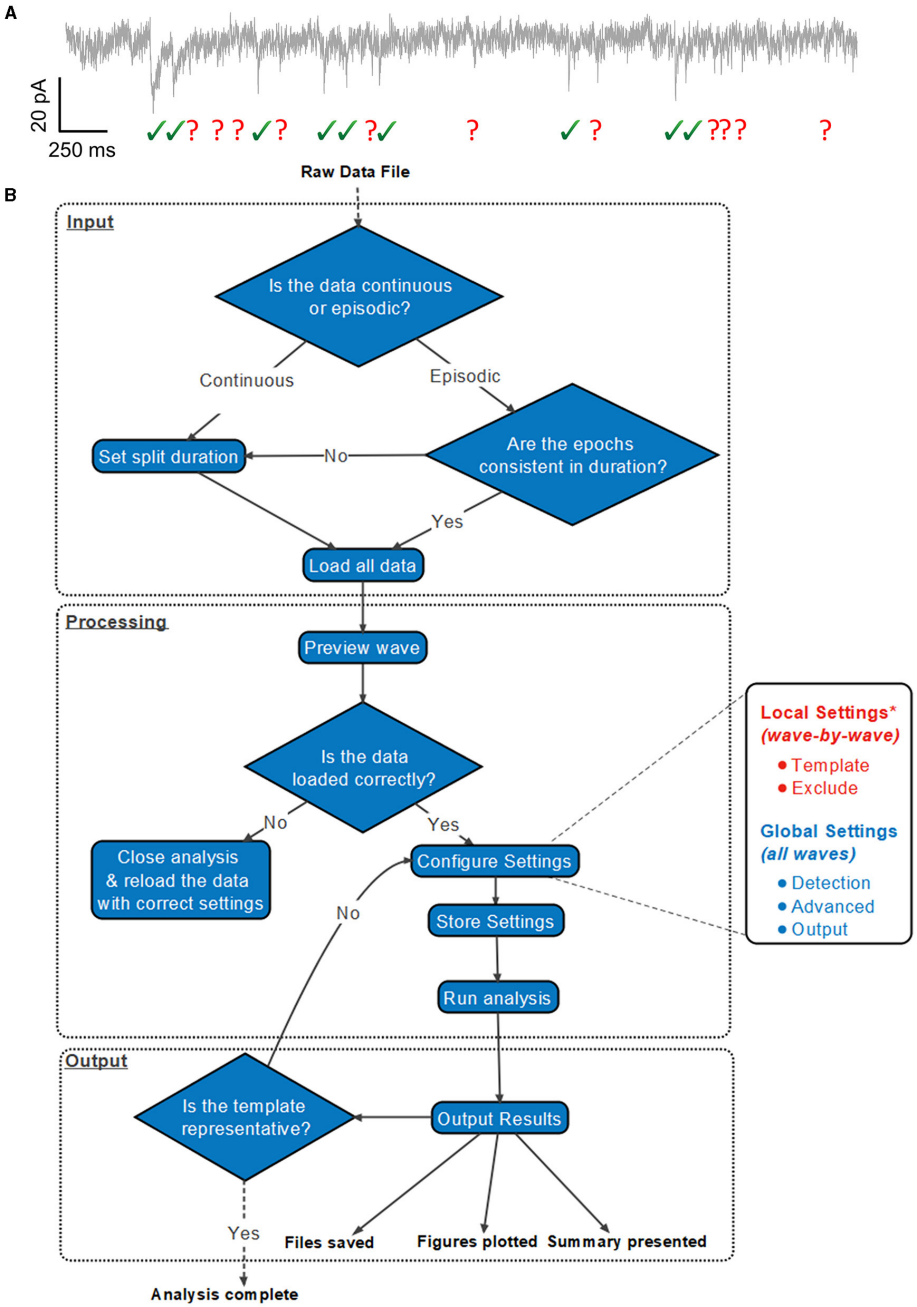
Exemplary analysis run workflow using Eventer. **(A)** Exemplary synaptic recording trace illustrating the difficulty in reliably manually selecting candidate events from the background noise. **(B)** Example analysis run illustrates how users are anticipated to work with Eventer. Work sections are largely split into three distinct regions: input, processing, and output. During the input, a raw data file must be selected, and a decision must be made to split the data if necessary. In the processing stage, local and global settings can be adjusted before running the analysis. Data are then presented and saved in the output phase. It is then possible to iterate the analysis and refine the event template, or train a machine learning model and use this for the classification of candidates detected in test datasets. *Local settings can be applied to all waves, as per the global settings in the GUI, if desired.

A suggested workflow for working with Eventer is then presented in Figure 1. Before selecting the raw data file, the user is advised to consider whether the data file being loaded contains either continuous or episodic acquisition. Episodic data with each epoch consistent in duration, or continuous data, can then be split into waves. It is not possible to split the data once it's been loaded. Splitting a data file has several specific benefits that can all be considered part of the processing of data before analysis; otherwise, a continuous data recording is loaded as a single wave.

#### 1.1.2 Processing

Once a data file has been loaded, Eventer will load a preview of the first wave, whether or not the recording is split. This preview allows the user to determine if the data have been correctly loaded. If not, the data should be reloaded; otherwise, the user can proceed to configure the analysis settings. Users can change the local, wave-by-wave, or global settings, which apply to all waves.

**Figure 2:**
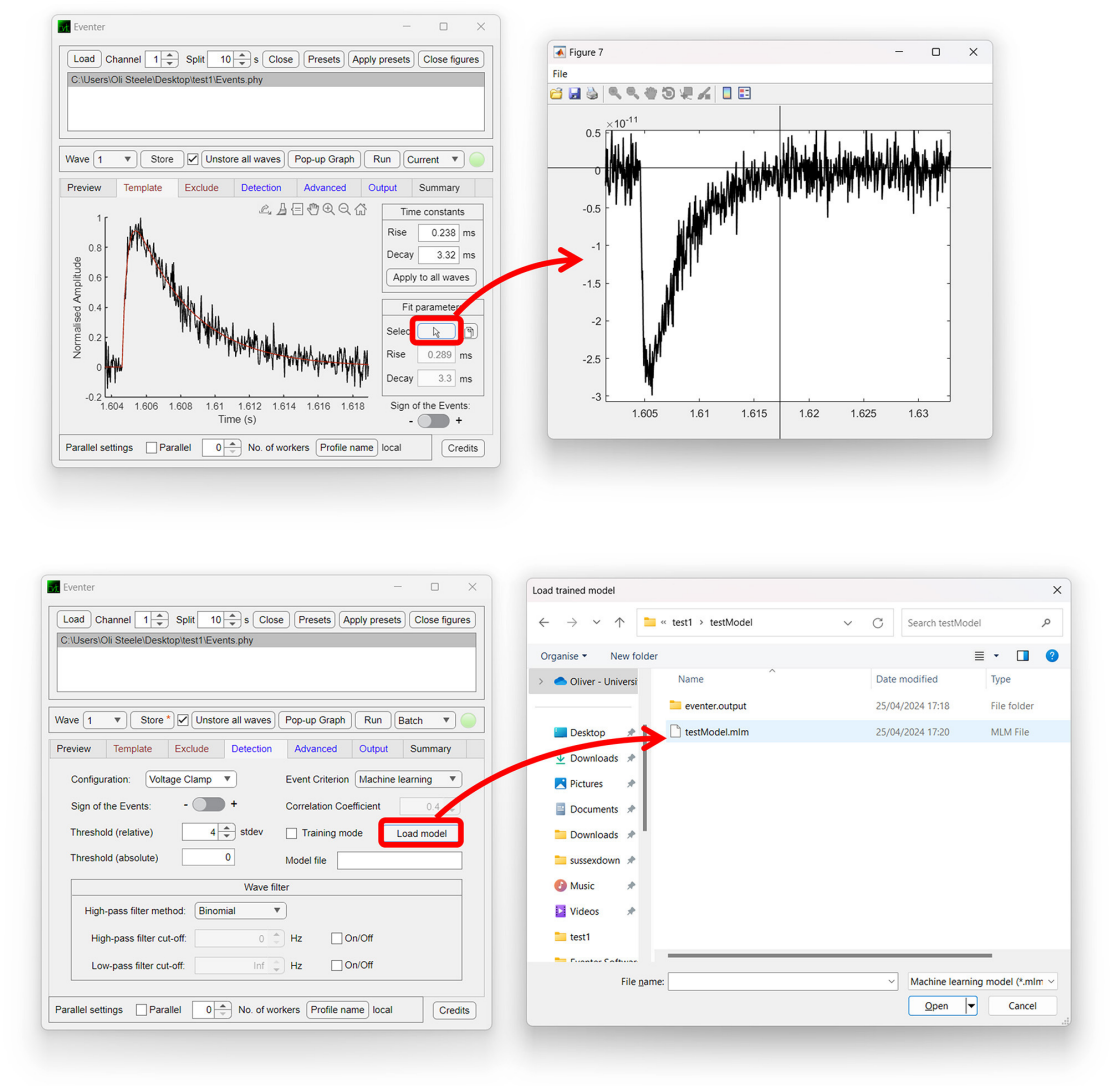
Easy-to-use graphical user interface of Eventer event detection software. Eventer features a clean, easy-to-use graphical user interface. **(Top)** The template panel is shown here, where users can select exemplary events to generate their parameters from. **(Bottom)** The detection panel is shown here, where users can select different event criterion methodologies, such as machine learning and Pearson's, and also load saved models through the opening of a file dialog window.

Both the Template and Exclude waves are considered local settings, so changes made here only apply to the wave currently selected unless specified otherwise to apply to all waves. In the Template panel, a user can define time constants of the rise and decay of the synaptic event if they are known or bring up a pop-up window to allow time constants to be measured from an exemplary user-selected event as shown in Figure 2 (top). In the Exclude panel, users can instead choose to exclude regions of the data that they do not want to be included in the subsequent analysis. If that data are from continuous data and split to a value the same as the interval between repeating recording artifacts such as test pulses or stimuli, these can be excluded from the analysis here.

The global settings are outlined on the Detection, Advanced, and Output panels. The Detection panel allows the user to select settings relevant to event detection mode itself, including filtering methods, threshold level, and event criteria. The threshold allows users to specify a threshold, either set as standard deviations of the background noise of the deconvolution wave (i.e., detector trace), or an absolute threshold value of the deconvolution wave, for initial event detection. If the event criterion is set to Pearson's, a correlation coefficient can be set here also. If, however, the event criterion is set to machine learning, a previously trained model can then be loaded, as shown in Figure 2 (bottom); otherwise, training mode can be enabled, whereby users can train a machine learning model of their own. Note that no thresholding of the correlation coefficient is applied when using the machine learning approach, the Advanced panel includes several settings outlined further in the manual, available online (Eventer, 2022). Finally, the Output panel allows users to define several output-specific settings, such as the format to save figures and data in (Table 1).

If the user is happy with the settings and the settings are stored, the user can then choose whether to analyse the single wave or a user-selected batch of waves. It is also worth noting at this point that users can take advantage of the parallel processing capabilities included as part of Eventer to speed up analysis dramatically by accessing multiple cores at once.

#### 1.1.3 Output

Following the completion of the analysis, Eventer then displays a summary of the results in the Summary panel. Files, and figures, are also saved in the desired output format, as specified before (Table 1). Finally, a range of summary plots are then plotted for the user to rapidly interpret the output of analysis and the suitability of the template fit. It may be advised that if the fit is not appropriate here, adjust the time constants with those in the Summary section and iteratively re-run the analysis until the template fit is appropriate (Figure 1).

### 1.2 Eventer workflows: analysis

**Figure 3:**
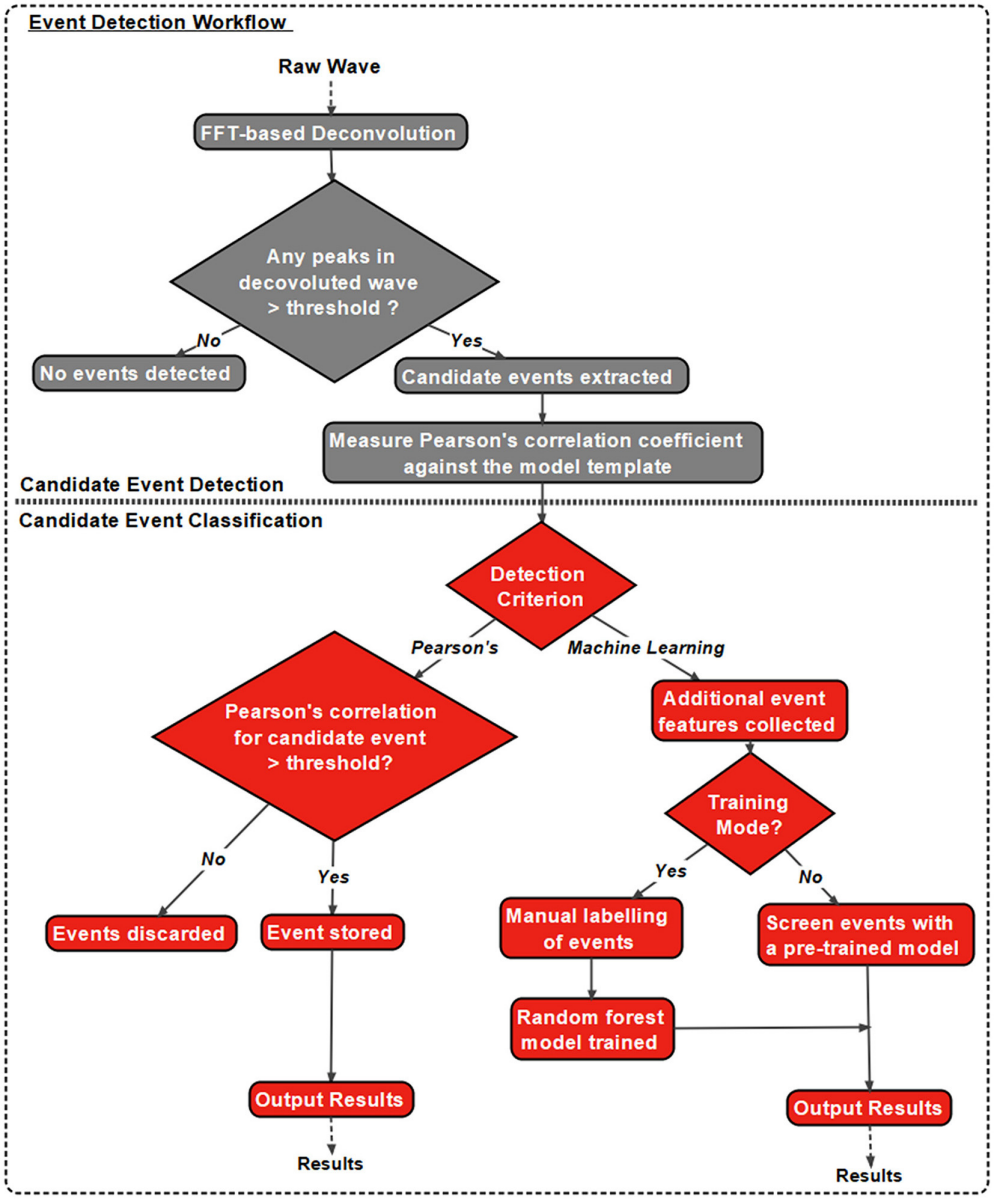
Eventer features a novel event detection protocol. Raw waves are initially deconvoluted by fast Fourier transform (FFT)-based deconvolution and compared to the event template. The resultant deconvolution wave (a.k.a. detector trace) depicts how similar, in the frequency domain, the data are at each point to the event template. If candidate events are above the user-defined candidate events are then made available for classification; otherwise, they are discarded. At this point, Pearson's correlation coefficient for each event against the model template is recorded. Next, dependent on the user-selected detection criterion, candidates will either be screened and saved if above the set Pearson's threshold and output as results, or additional event features will be computed if in machine learning mode. If in training mode, manual labeling of candidate events occurs to train a random forest model; otherwise, a pre-trained model can be loaded and passed over candidate events before outputting the results.

**Figure 4:**
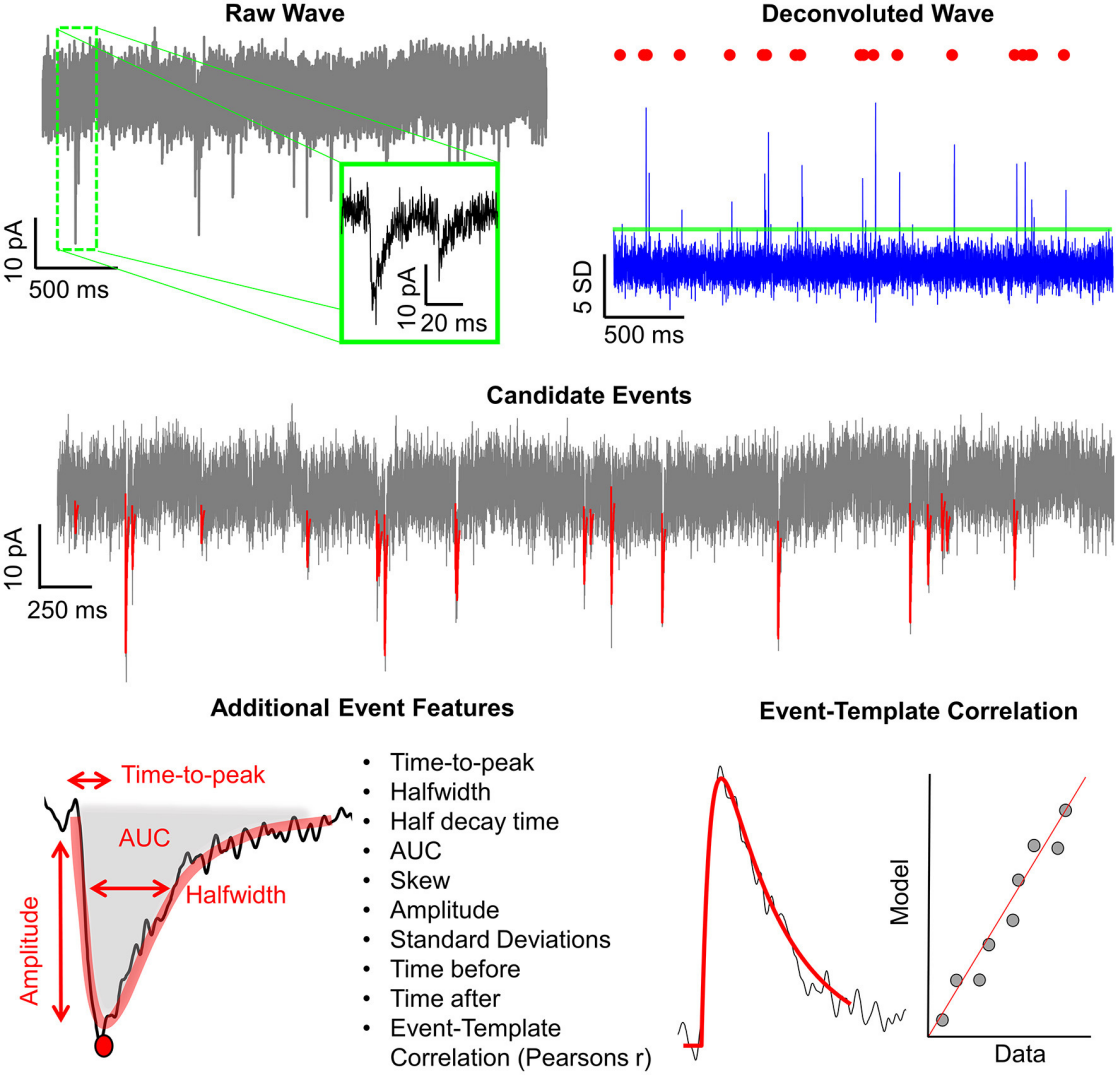
Graphical depiction of the analysis workflow. Shown here is a graphic representation of each of the relevant steps of Eventer-based event detection. Initially, a raw wave undergoes fast Fourier transform (FFT)-based deconvolution and comparison against the event template **(top)**, at which point candidate events are selected if they are above a set threshold **(middle)**. If set to machine learning mode, then additional event features are measured, including Pearson's correlation, and used to train a random forest model. Otherwise, when in Pearson's mode, if events have a Pearson's correlation coefficient below the set threshold, then these events are discarded before the results are output.

In addition to the intuitive GUI, Eventer utilizes a novel event detection methodology depicted in Figures 3, 4. Eventer initially performs FFT-based deconvolution, and then in the frequency domain, the resultant deconvolution wave is a measure of similarity to the event template at each point of the recording. The time points of peaks in the deconvolution wave exceeding a set threshold, expressed either as an absolute value, or a scale factor of standard deviations of the noise, indicates the start times of candidate events. A Pearson's correlation coefficient is then calculated for each candidate event with respect to the event template. Eventer will then either declassify events with a Pearson's correlation coefficient below a user-defined threshold (between −1 and +1) if the event criterion is set to Pearson's before outputting results, or measure additional event features (depicted in Figure 4) if set to Machine Learning mode.

In Machine Learning mode, the user has the option to specify whether this run executes in training mode, whereby the user will then be provided with a pop-up window to manually label candidate events selected by the FFT-based deconvolution. The user's classification will then be used to train a random forest machine learning model, which could then be run over new data. If not in training mode, a previously trained model can be loaded and run, with events being screened with the preferences included implicitly in the trained model.

## 2 Methods

### 2.1 Simulated events

#### 2.1.1 Accuracy

**Figure 5:**
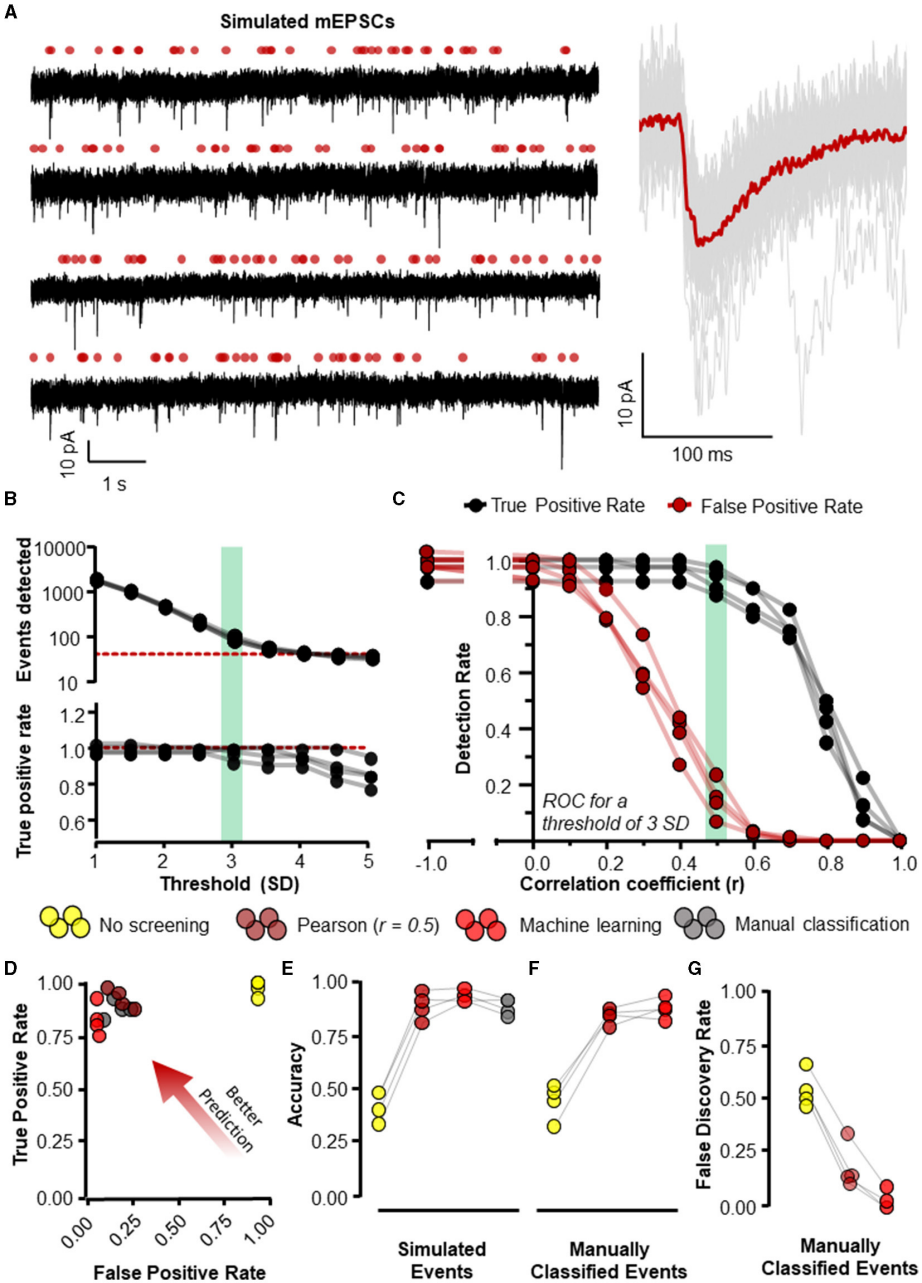
Eventer accurately detects synaptic events. Eventer was able to accurately classify simulated events, either those that were simulated or those that were classified by manual selection. **(A)** The four waves represent simulated events (of variable amplitude and time course) added to real whole-cell recording noise and used as data to assess the accuracy of Eventer (*left*). The *right* of the panel shows the mean ensemble average of the 40 simulated events in red plotted over the individual events in gray from the first simulated wave. **(B)** (*top*) The total number of events detected in each of the simulated waves decreases as the threshold of the signal in the deconvolution wave increases. The red dotted line represents the number of simulated events in the waves **(bottom)**. The true positive rate of event detection decreases as the threshold of the deconvolution wave is increased above 3. **(C)** The true positive rate (TPR) shown in black and the false positive rate (FPR) shown in red are plotted as a function of the correlation coefficient (r) in Eventer with the threshold of the deconvolution wave set at 3 standard deviations. **(D)** Graph showing how the results of different classification methods occupy ROC space, with scores in the bottom-right indicating poor prediction/classification (through high false positive and low true positive) and top-left indicating better prediction/classification (through low false positive detections but high true positive detections). **(E)** Depicts an overall score of accuracy of Eventer to select simulated events. **(F)** An overall score of accuracy by comparing the events selected by each parameter relative to manual classified events. **(G)** The main benefit of Eventer machine learning is a large reduction in the false discovery rate when trying to reproduce manual classification of synaptic events. Statistical results for this figure are documented in Table 2.

Data used for the accuracy tests presented in Section 3.1 and Figure 5 were generated by adding simulated miniature excitatory postsynaptic current-like waveforms to real whole-cell recording noise using the custom “*simPSCs_recnoise.m*” script and the “*noiseDB.abf* ” data file, which are all available online at the acp29/Winchester_EVENTER repository on GitHub (Penn A., 2024). The background recording noise from CA1 pyramidal neurons in organotypic hippocampal slices (see Methods section in Elmasri et al., 2022a,b) was acquired with a MultiClamp 700B amplifier (Molecular Devices), low-pass filtered (4 kHz, low-pass Bessel filter), and digitized (40 kHz) with a USB-X Series Multifunctional DAQ interface (NI USB-6363, National Instruments) interfaced through python-based, open-source data acquisition software, ACQ4 software (v0.9.3) (Campagnola et al., 2014). The salt compositions of extracellular and intracellular solutions are described in Elmasri et al. (2022a,b). Ionotropic glutamatergic and GABAergic channels were pharmacologically inhibited with (in μM): 10 NBQX, 50 APV, and 10 Gabazine, respectively, to isolate background noise during whole-cell recordings (e.g., stochastic ion channel openings, instrument noise, etc.).

For each of the eight simulations generated, 40 events were individually simulated using randomly sampled amplitude and kinetics and added to 9.9 s of whole-cell recording noise. The procedure to simulate each event went as follows: A value for the amplitude (in -pA), rise time constant, and decay time constant (both in ms) were generated by exponential transformation of the random number sampled from a normal distribution (with mean and standard deviation) of *N*(2.46, 0.35), *N*(−0.31, 0.60), and *N*(1.48, 0.46), respectively. These distribution parameters were chosen based on the log-normal distributions of miniature excitatory postsynaptic current (mEPSC) parameters obtained when analyzing recordings of CA1 neurons (without NBQX and with 1 μM Tetrodotoxin) in our lab. The only other constraint on the parameters of the simulated events was that the sampling for the decay time of the event was repeated if its time constant was less than or equal to its rise time constant. The rise and decay time constants were used to generate a synaptic-like waveform using the sum-of-two exponential model and then peak-scaled (Roth and van Rossum, 2009). The sample point for the event onset was drawn from a random uniform distribution across the total number of simulation samples (396,000) and represented as a value of 1 in a vector of zeros. No limits were placed on how close events could be to each other. The event waveform was then simulated at the random time of onset by fast Fourier transform (FFT) (circular) convolution using the equal-length vectors defining the event onset and the synaptic-like waveform. The resulting vector was scaled by the event amplitude and added to the equal-length vector of whole-cell recording noise. The procedure was repeated 40 times in total for each of eight different whole-cell noise and with different random seeds. To analyse the simulations with the machine learning method in Eventer, half of the simulated waves (*n* = 4) were used exclusively for training four different machine learning models, and the other half of the waves (*n* = 4) were analyzed and used exclusively for the accuracy evaluation tests.

Four different classification methods were compared: Pearson's correlation coefficient (r) threshold of 0.5, machine learning using random forests, manual classification by an expert user, and no screening/classification (i.e., *r* = −1). Other non-default settings used for analyses the conditions were rise and decay time constants of 0.44 and 6.12 ms for the template, and a detection threshold of three times the standard deviation of the noise in the deconvolution wave, which was filtered with high- and low-pass cutoffs of 1 and 200 Hz.

The Matlab function “*ismembertol*” was used to identify matching times of event onset (within 1.2) for events that were detected and/or classified vs. those that were originally simulated (Figures 5B–E) or manually classified (Figures 5F, G). With this information, the following receiver operating characteristics (ROC) were computed. A true positive (TP) was determined as a detection classified as true that was indeed a simulated event. A false positive (FP) was a detection classified as a true event but was not. A true negative (TN) was a false detection correctly classified as false, whilst a false negative (FN) was where simulated events were incorrectly classified as false. From these values, it was then possible to calculate the following metrics. First, a false positive rate (FPR), interpreted as the rate at which Eventer incorrectly classifies an event to be a true synaptic event, was calculated as follows;


$$\begin{array}{l} False\;positive\;rate\;\left(FPR\right)=\;\frac{FP}{FP+TN} \end{array}$$

True positive rate (TPR), interpreted as the rate at which Eventer correctly classified an event as a true synaptic event, was calculated as follows:


$$\begin{array}{l} True\;positive\;rate\;\left(TPR\right)=\frac{TP}{TP+FN} \end{array}$$

An overall measure of accuracy was then calculated as follows:


$$\begin{array}{l} Accuracy=\;\frac{TP+TN}{TP+TN+FP+FN} \end{array}$$

Finally, a false discovery rate (FDR), interpreted as the proportion of all of the events classified by Eventer as true events that are in-fact not events, was calculated as follows:


$$\begin{array}{l} False\;discovery\;rate\;\left(FDR\right)=\;\frac{FP}{FP+TP} \end{array}$$

The computations are documented in a script, “invcompE.m,” which is available online at the acp29/Winchester_EVENTER repository on GitHub (Penn A., 2024).

#### 2.1.2 Speed and parallel computing performance

**Figure 6:**
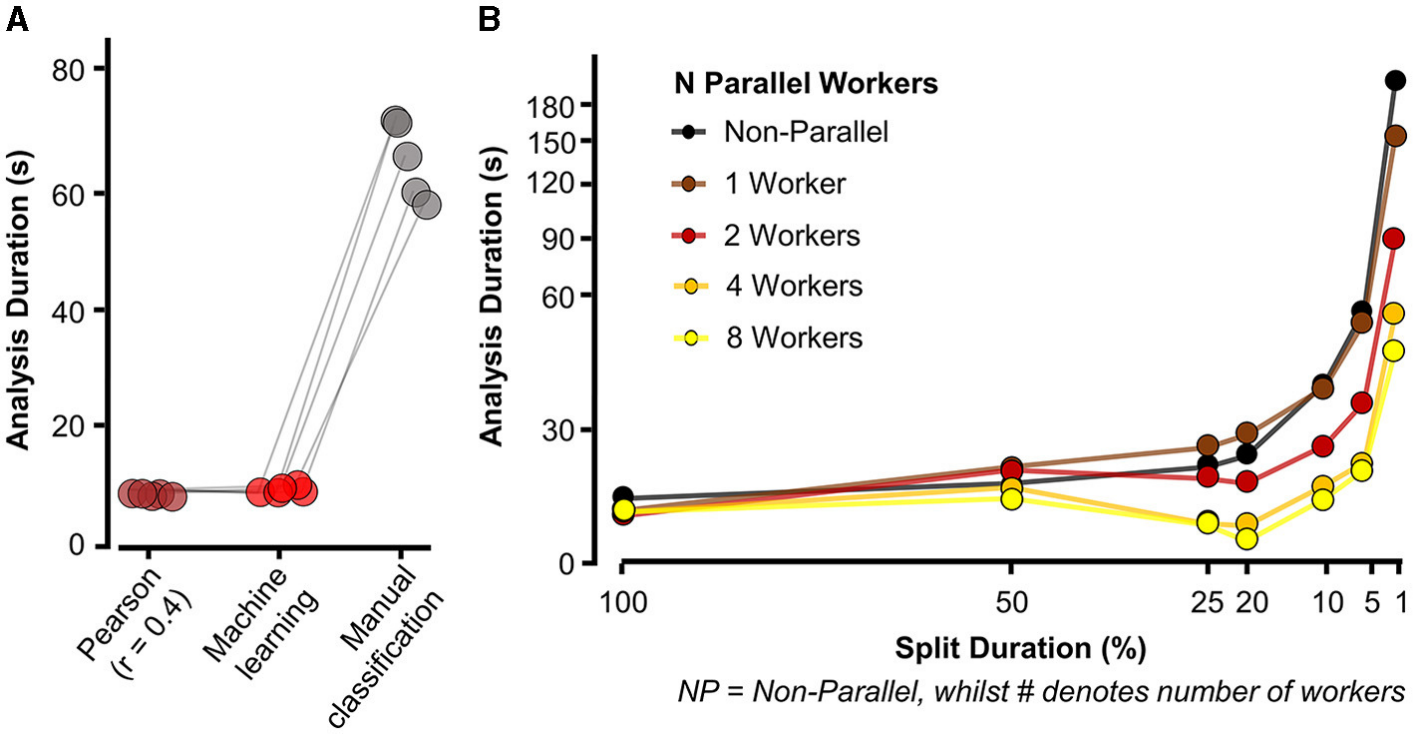
Eventer increases speed of synaptic event detection via parallel processing. **(A)** Eventer with machine learning or Pearson's correlation coefficient threshold for classification was considerably faster than manual classification. **(B)** Speed (in seconds) taken for analysis to complete and display results for a 100-s-long recording with simulated events occurring at a frequency of 3 Hz on top of physiological recording noise, with multiple split percentages and numbers of parallel workers. *X*-axis plotted on logarithmic scale. Split duration is plotted in reverse order, with the left (100%) indicating no split and the further right suggesting an increasing degree of splitting the data. For example, 5% split of 100 s would equate to 20 × 5-s waves being generated.

The performance test in Figure 6A was performed on the simulated waves, as detailed in Methods 3.1.1 and illustrated in Figure 4A. Here, the simulated waves were then analyzed with three detection methods: Pearson, machine learning (pre-trained model), and manual classification of events, in which a threshold of 3 standard deviations and a Pearson's r of 0.4 were set and used across all repeats (five per condition). The total time taken for Eventer to complete detection was then recorded in seconds.

Simulated events used for the performance tests in Figure 6B were generated with white noise (RMS = 2 pA) using the “*simPSCs.m*” script, which is available at the acp29/Winchester_EVENTER repository online on GitHub (Penn A., 2024). We simulated a single 100-s wave containing 300 events from log-normal distributions for amplitude, rise time constant, and decay time constant with a mean of 20 pA, 0.4 ms, and 4 ms, respectively, and a coefficient of variation of 0.5 (i.e., 50%). Inter-event intervals were constrained to have a proximity of no <1.5 ms. To evaluate performance using different settings in Eventer, the 100-s simulation was split with the following denominations (in seconds): 1, 5, 10, 20, 25, and 50, which were then plotted as percentages of the total time. The number of workers (i.e., physical cores) dedicated to parallel processing was then changed from 0 (non-parallel, with figure plotting) to 1 (non-parallel, but without figure plotting), 2 (two parallel physical cores without figure plotting), 4 (four parallel physical cores without figure plotting), and 8 (eight parallel physical cores without figure plotting). The time taken to analyse the data (from selecting “run” to the display of summary results) was then recorded in seconds and plotted against the recorded split percentage and number of workers. All events, across all conditions, were detected with the same data and template settings, although, other than the number of events, our experience is that the properties of the events and the noise have relatively minor impact on the performance of Eventer. The computer used for testing Eventer parallel performance had a Ryzen 7 3800X 4.2 GHz 8 core processor with 32GB DDR4 3200 MHz random access memory (RAM), 1TB non-volatile memory express (NVMe) solid-state drive (SSD), and was operated using Windows 11.

#### 2.1.3 Consistency

In the third experiment assessing the consistency of analysis between users, analysis was conducted on real recordings of mEPSCs from CA1 neurons in organotypic hippocampal slices, in which voltage-gated sodium channels were inhibited with 1 μM tetrodotoxin and ionotropic GABAergic channels were pharmacologically blocked with 50 μM picrotoxin. Organotypic hippocampal brain slices were prepared using methods and with ethical approval as described previously (Elmasri et al., 2022a,b). Recordings were performed with a cesium methanosulfonate-based intracellular recording solution containing the following (in mM): 135 CH_3_SO_3_H, 135 CsOH, 4 NaCl, 2 MgCl_2_, 10 HEPES, 2 Na_2_-ATP, 0.3 Na-GTP, 0.15 C_10_H_3_ON_4_O_2_, 0.06 EGTA, and 0.01 CaCl_2_ (pH 7.2, 285 mOsm/L). Artificial cerebrospinal fluid (aCSF), perfused over organotypic hippocampal slices at 3 ml/min during recordings, was maintained at 21°C, balanced to ~305 mOsm/L, and bubbled with 95% O_2_/5% CO_2_ continuously, contained the following (in mM); 125 NaCl, 2.5 KCl, 25 NaHCO_3_, 1.25 NaH_2_PO_4_, 1 MgSO_4_.7H_2_O, 10 D-glucose, 2 CaCl_2_, and 1 sodium pyruvate (pH 7.4). Three 30-s recordings were analyzed, each by a separate group of five postgraduate neuroscience students (15 in total) who had a basic familiarity of synaptic event detection. Each student was asked to select event parameters and manually classify events, and the details of their individual classification were compared with the same for each of the other students within their group by calculating Matthew's correlation coefficients (MCC).

The equation for the Matthew's correlation coefficient is illustrated below:


$$\begin{array}{l} MCC=\;\frac{TP\times \;TN-FP\times TN}{\sqrt{\left(TP+FP\right)\times \left(TP+FN\right)\times \left(TN+FP\right)\times \left(TN+FN\right)}} \end{array}$$

All students were then given a single model and asked to pass this model over the same recording without changing their chosen event parameters, and the comparisons using MCC were repeated.

#### 2.1.4 Statistics

**Figure 7:**
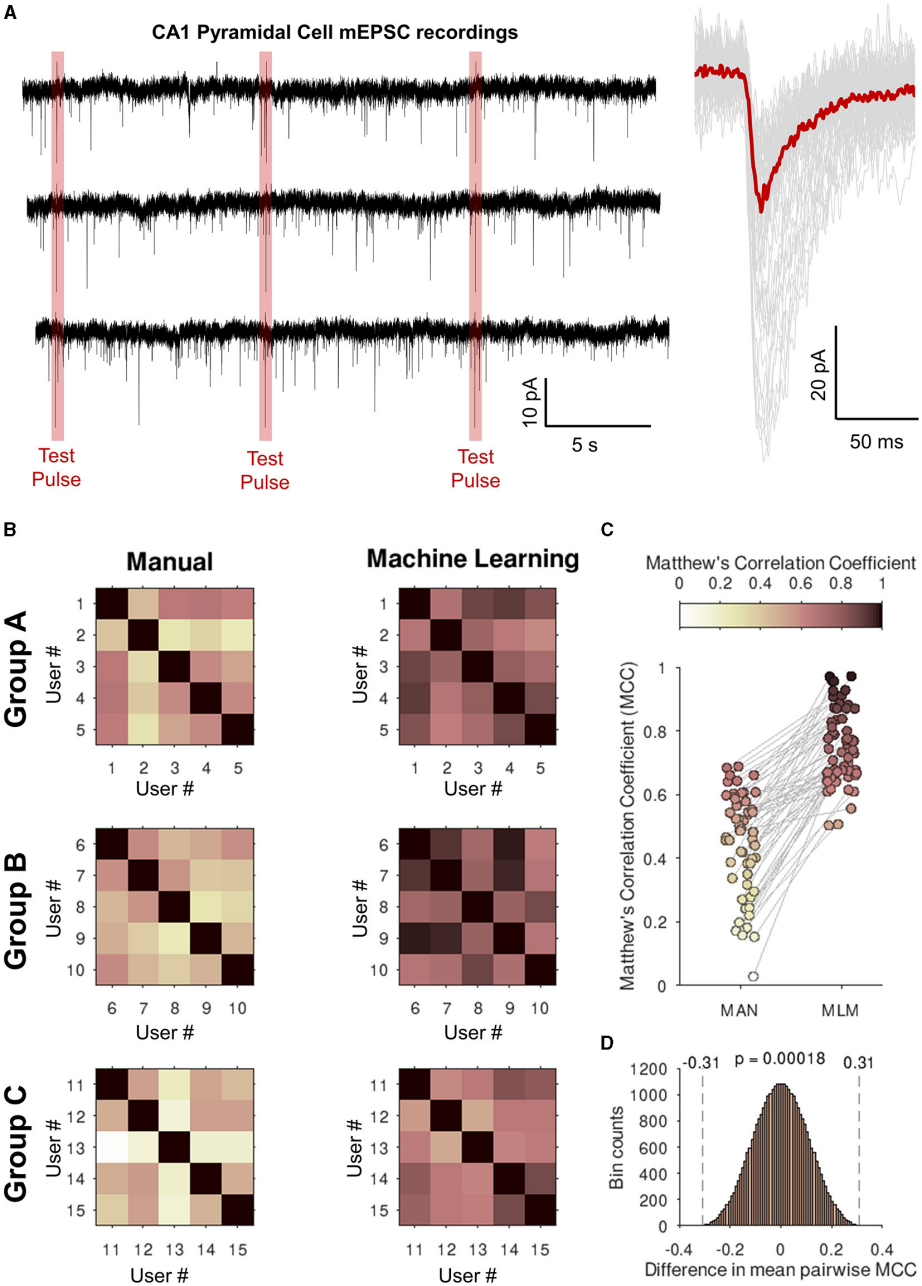
Eventer increases consistency of analysis between users when classifying synaptic events with a single machine learning model. **(A) (left)** Whole 30-s traces of miniature excitatory postsynaptic currents (mEPSCs) recorded from CA1 pyramidal neurons in organotypic hippocampal slices that students in the groups below (Groups A–C). Highlighted in red are the voltage step test pulses to observe changes in access resistance that were excluded from the analysis. **(Right)** Individually detected events are shown in gray with the ensemble average overlaid in red. **(B)** Three groups (Groups A–C) of five students were asked to detect and classify events as either true events or false positive detections. Shown in the upper-right half of the grids Matthew's correlation coefficients (MCCs) are plotted against the users' own classifications for the intra-group pairwise comparisons (left, Manual). The users then all loaded a machine learning model generated by a single expert user and then re-ran the event classification (right, machine learning). **(C)** Values of the off-diagonal MCC scores are plotted for each pair of student comparisons. **(D)** Permutation distribution for the difference in MCC scores between manual and machine learning classification.

Graphs in figures were created either in GraphPad Prism (version 8) or GNU Octave (version 8.3). The Statistics-Resampling package (version 5.5.10) in GNU Octave was used to perform null hypothesis significance testing, specifically using the “bootlm” (for the tests in Figure 5) and “randtest2” (for the tests in Figure 7) (Penn A. C., 2024). Using “bootlm,” *p*-values for two-way ANOVA without interaction were calculated by wild bootstrap resampling of the residuals (with Webb's six-point distribution). With the same function, *post-hoc* test *p*-values for pairwise differences were computed using the studentized bootstrap-*t* method, and the family-wise type 1 error rate was controlled using the Holm–Bonferroni step-down correction. The confidence intervals reported in Table 2 by “bootlm” are asymmetric studentised 95% confidence intervals after wild bootstrap. For the permutation test in Figure 7, the “randtest2” function with *paired* argument set to true was used to permute the allocation of the sets of event times for each subject between the manual and machine learning conditions and calculate a two-tailed *p*-value from the permutation distribution of intra-group pairwise differences in Matthew's correlation coefficient. See the Data availability statement for information on how to access and reproduce the statistical analysis. All statistics reported in the text are mean ± SD.

**Table 2:** Statistical results for Figure 5.

**Comparison**	**Mean [lower CI, upper CI]**	***p*-Value**	**Sig**.
**Classification of simulated events (** **Figure 5E** **)**			
No screening – Pearson's (*r* = 0.5)	−0.457 [−0.520, −0.393]	<0.001	^***^
No screening – machine learning	−0.498 [−0.559, −0.437]	<0.001	^***^
No screening – manual classification	−0.451 [−0.533, −0.369]	<0.001	^***^
Pearson's (*r* = 0.5) – machine learning	−0.042 [−0.083, −0.001]	0.145	ns
Pearson's (*r* = 0.5) – manual classification	+0.006 [−0.068, +0.080]	0.883	ns
Machine learning – manual classification	+0.048 [−0.030, +0.123]	0.452	ns
**Accuracy of classified events compared to manually classified events (** **Figure 5F** **)**			
No screening – Pearson's (*r* = 0.5)	−0.399 [−0.466, −0.331]	<0.001	^***^
No screening – machine learning	−0.437 [−0.531, −0.342]	<0.001	^***^
Pearson's (*r* = 0.5) – machine learning	−0.038 [−0.105, +0.030]	0.364	ns
**False positive rate of identifying manually classified events (** **Figure 5G** **)**			
No screening – Pearson's (*r* = 0.5)	+0.354 [+0.293, +0.416]	<0.001	^***^
No screening – machine learning	+0.513 [+0.445, +0.579]	<0.001	^***^
Pearson's (*r* = 0.5) – machine learning	+0.159 [+0.085, +0.233]	<0.001	^***^

The mean differences are summarized along with 95% bootstrap-*t* confidence intervals. The *p*-values for the mean difference are from *post-hoc* tests using the Holm–Bonferroni method (see Methods). All detection used a lower threshold of 3 SD for the initial FFT-based deconvolution. ^***^p ≤ 0.001.

## 3 Results

### 3.1 Eventer enables accurate detection and classification of synaptic events

To evaluate the accuracy of Eventer for the detection and classification of synaptic-like waveforms, we simulated recordings, each consisting of 40 events generated by FFT convolution and added to a unique 9.9-s segment of recording noise measured by whole-cell patch clamp in CA1 neurons whilst pharmacologically blocking synaptic receptors (see Section 2.1.1 for details). The root-mean-square deviation (RMSD, calculated in rolling 100 ms windows) of the recording noise was 2.43 pA (*SD* = 0.085 pA), and the log-normal distributions used for random sampling for the amplitudes, rise time constants, and decay time constants for the events had a mode [2.5%−97.5% percentiles]: 10.4 [5.9–23.2] (–)pA, 0.51 [0.23–2.38] ms, and 3.55 [1.78–10.82] ms, respectively. A further four independent simulated recordings were created for the purposes of training machine learning models (Figure 5A).

Event detection in the simulated waves was conducted with the multiple modalities of event detection and classification that exist in Eventer. The most rudimentary form uses threshold-crossing of the detector trace after FFT-based deconvolution (Pernía-Andrade et al., 2012), without any further screening or classification (i.e., switching off Pearson's correlation coefficient in Eventer to −1). In order to compare classification methods, we first established a detection threshold for the deconvolution, that is sensitive enough to detect all (or most) of the simulated events whilst also providing opportunity for alternative screening or classification of candidate events. The number of events detected dropped steeply as the detection threshold was raised from 1 to 3 standard deviations of the noise, and more slowly for thresholds of 3 to 5 standard deviations (Figure 5B). However, a decline in the rate of true positive event detections became increasingly apparent at thresholds of about 3 and above (Figure 5B). Whilst higher thresholds for deconvolution (e.g., 4 SD) can provide good accuracy in the tests we describe hereafter (Pernía-Andrade et al., 2012), they do not yield a sufficient number of candidate events for further tuning the classification process. Therefore, a threshold of 3 standard deviations (SD) was used for the subsequent tests. Eventer can compare candidate events detected by the FFT-based deconvolution, discarding those events where the Pearson's correlation coefficient drops below a set threshold. We found that setting higher thresholds for the correlation coefficient reduces the number of false positive classifications, albeit at the cost of missing true positive events (Figure 5C). Indeed, the TPR dropped rapidly as the threshold for the correlation coefficients exceeded 0.5 (Figure 5C). We next examined the receiver operating characteristics (ROC) with these settings (3 SD with or without *r* = 0.5) compared with those using a detection threshold of 3 SD and manual or machine learning event classification.

Manually classifying candidate events after deconvolution resulted in very similar FPR and TPR scores compared to screening using the Pearson's correlation coefficient threshold of 0.5 (Figure 5D). However, the threshold of *r* = 0.5 may not always be optimal for event screening in any given context and could vary depending on the variability of event time course kinetics and signal-to-noise ratio. A benefit of using Eventer is the option to reproduce the accuracy of manual event classification by using a trained machine learning model; thereby, users can avoid arbitrarily setting a threshold for the matching of the template time course with the events. To test the machine learning capabilities of Eventer, four machine learning models were generated by training four independent waves created using different segments of background noise recordings and the same data-generating process as the test simulations with different random seeds (see Section 2.1.1). Each model was trained on 83–107 candidates (detected by the FFT-based deconvolution), of which 37–40 represented the originally simulated events. The out-of-bag classification error for the trained models, which is used to estimate the prediction error of the random forests, was typically ~5%, suggesting that even using relatively little data to train the models, reasonable predictions could be made for classification. Each of the original four test simulations was then analyzed with a different machine learning model, and ROC metrics were calculated and compared with the other detection and screening/classification methods. The similar ROC metrics suggest that using Eventer with machine learning correctly classifies events at a comparable rate to manual event classification by an expert user without the need to define an arbitrary threshold (Figure 5D).

The ROC metrics of correct and incorrect event detection can be quantified as a singular accuracy score as defined in Methods 3.1.1 and illustrated in Figure 5E. Event detection with no additional screening of events detected using a low threshold (3 SD) of the deconvolution signal is the least accurate event detection method (0.43 ± 0.070). Additional screening using a threshold of 0.5 for Pearson's correlation coefficient increases the overall accuracy of detecting simulated events (0.88 ± 0.062), which is to a similar extent as manual event selection (0.88 ± 0.039) in this example. Importantly, the machine learning-based event classification seemed to be similar in accuracy as manual event detection by an expert user (0.92 ± 0.029). The classification methods differed significantly in their accuracy for identifying simulated events [*F*_(3, 9)_ = 119.3, *p* < 0.001, Table 2] after accounting for overall differences between the simulations (bootstrapped two-way ANOVA without interaction). *Post-hoc* pairwise comparisons indicated that the accuracy of detecting simulated events was significantly different from the group with no screening or classification of events (*p* < 0.001, Holm–Bonferroni tests). However, we could not resolve differences between the other classification methods, suggesting that they are all capable of performing similarly well (*p* > 0.05, Holm–Bonferroni tests). These data suggest that the machine learning capabilities included in Eventer make it comparably accurate in the detection of simulated events for the expert human user (when trained by the expert user).

To confirm whether the same events were being detected by the different detection methods and the expert user, and thus act as an effective substitute for manual selection, the individual events detected by each detection method were then compared with the events detected by the expert user (Figure 5F). No screening produced the lowest accuracy scores (0.45 ± 0.084). Screening with the threshold of 0.5 for Pearson's correlation coefficient increased accuracy considerably (0.85, ± 0.037), only just shy of the accuracy for classification with a machine learning model (0.89 ± 0.049). There was a significant difference in accuracy across the various methods in their ability to identify manually classified events [*F*_(2, 6)_ = 80.9, *p* < 0.001, bootstrapped two-way ANOVA without interaction]. However, *post-hoc* pairwise tests showed that only the comparisons to the group with no event screening/classification were significantly different (*p* < 0.001, Holm–Bonferroni tests). Examination of the ROC metrics indicated that properties of these classification methods did differ in some respects, notably in their false discovery rates [*F*_(2, 6)_ = 179.7, *p* < 0.001, Bootstrapped two-way ANOVA without interaction] with statistical significance for all pairwise comparisons (*p* < 0.001, Holm–Bonferroni tests). Not surprisingly, detection without screening had the highest false discovery rate (0.55 ± 0.084), whereas classification using a threshold for the correlation coefficient of 0.5 (0.19 ± 0.109) was ~4 times higher than for the machine learning model (0.03 ± 0.046) (Figure 5F). These data suggest that Eventer is effective at acting as a substitute for manual expert user selection.

Taken together, these results suggest that not only is machine learning-based event detection with Eventer reliable at correctly classifying simulated synaptic events without the requirement of tuning an arbitrary threshold, but Eventer is also able to reliably reproduce the event selections of an expert user.

### 3.2 Eventer increases the speed of synaptic event detection

For Eventer to be used in place of manual event selection, it would also need to reduce the time taken to perform analysis. To test the speed of Eventer relative to manual event selection, simulated recordings were then analyzed using Eventer with both Pearson's screening and a machine learning model, and the amount of time taken was measured (see Section 2.1.2). This duration was then compared to the amount of time taken to manually classify the same number of events. Figure 6A illustrates the time taken to analyse recordings across these conditions. The mean amount of time taken for Pearson's detection (5.87 ± 0.228 s) and machine learning detection (5.84 ± 0.567 s) in Eventer were comparable and dramatically lower than the time taken to manually classify the same number of events (63.3 ± 6.663 s). These data suggest that not only can Eventer reliably reproduce the selection of an expert user, but it can also do so in a tenth of the time. In this experiment, parallel processing was not used to be as comparable as possible for other users. Utilizing parallel processing could further increase the speed benefits when data are required to be split into epochs for analyzing and summarizing changes in synaptic properties over time.

To illustrate how splitting the data and using parallel processing influence the speed of event detection, Eventer then detected events in a 100-s-long recording with 300 simulated events. Figure 6B (black trace, NP) then illustrates the amount of time taken to complete the event detection with various split times as a percentage of the total recording length in the absence of parallel processing. Here, it is perhaps fastest to not split the data (100 s analyzed, 300 events detected in 18.61 s), and inappropriate splitting of the data to 1% of its length (i.e., 100 × 1-s-long waves) dramatically increases the time taken (192.28 s). However, increasing the number of parallel workers increases the speed of analysis, regardless of the split duration. When increasing the number of parallel workers to 8, the speed of event detection dramatically increased from 192.86 s to 42.69 s when the split duration remained at 1%. Indeed, it is possible to fine-tune the number of parallel workers and split duration in Eventer. As shown here, Eventer was able to detect 300 events in 100 s worth of recording in 14.69 s when utilizing eight parallel workers, and the data are split to 20% of the total length. Taken together, these data suggest that Eventer is not only able to reliably and accurately classify synaptic events but also to do this faster than manual event classification.

### 3.3 Eventer increases the consistency of synaptic event detection between users

Eventer has been shown to decrease the time taken for event classification and to be accurate in doing so, relative to manual event detection. However, the other major issue remaining with the detection of spontaneous synaptic events is that detection methods between labs and users are inconsistent. By training a machine learning model in Eventer, it is then possible for other users to use this model to analyse their data. It is hypothesized that doing so should increase the consistency of event detection between users.

To test whether using a single machine learning model will increase consistency between users, three groups (A, B, and C) of five students were supplied with a simulated recording and asked to define their event templates and manually (MAN) classify events. All students were at a similar stage of education (postgraduate research) and were provided with equal amounts of training prior to starting classification. The students” classifications were then compared to each other within groups, and a Matthew's correlation coefficient (MCC) was generated to assess similarity. Immediately after this, the students were given a single machine learning model (MLM) trained by an expert user and asked to re-run their analysis with this model instead of their own without changing their selected event time constants. These values were then compared within the group again, and another set of MCCs was generated. Figure 7A then plots these MCC grids. A darker color here represents a correlation in the classification. Excluding self-comparisons (i.e., the diagonals of the MCC grids), the classifications of users were consistently more like one another when using a single machine learning model (MCC = 0.76) than without (MCC = 0.45), as shown in Figure 7B. Since the MCCs represent pairwise comparisons between users, the MCCs themselves are correlated and therefore violate the assumption of independent observations made by most common statistical tests. To evaluate whether the observed +0.31 improvement in the MCC by machine learning is statistically significant, we created and analyzed 2^15^ unique replicate samples by exchanging sets of the detected event times between the MAN and MLM conditions for each user to simulate the sampling distribution of the difference in the MCC under the null hypothesis (Figure 7C). The probability of observing an absolute difference in MCC at least as large as the observed 0.31 under the null hypothesis was 0.00018. This significant difference between manual and machine learning classification of synaptic events strongly suggests that using a single machine learning model can increase the consistency of analysis between users.

## 4 Discussion

The data presented provide proof of concept that Eventer is capable of accurately and correctly classifying candidate synaptic currents at a comparable level to that of an expert human researcher. Moreover, Eventer achieves the classification task far more rapidly than any human, and in a way that is reproducible between different users. In the current section, we will discuss the results in the context of alternative, existing approaches.

### 4.1 Alternate classification approaches

Eventer, which was first officially released in 2020 (Winchester et al., 2020), is not the only event detection software now to utilize machine learning, although it is one of the few to provide a graphical user interface. Zhang et al. (2021) developed software to detect excitatory postsynaptic potentials *in vivo* that they term machine learning optimal-filtering detection procedure (or MOD). This approach utilizes the Wiener-Hopf equation to learn the optimal parameters for a Wiener filter that can subsequently be applied to data to detect EPSPs. Since the MOD approach makes the assumption that synaptic currents will summate linearly, it is not clear to what extent the method will be robust to the presence of non-linearities, for example, introduced by voltage escape during the near coincident activation of nearby synapses. The random forest approach of Eventer is inherently more robust to such complex non-linearities due to its ensemble learning nature, and the initial detection of the candidates tolerates overlap, and some variation in the time course, of the synaptic waveforms (Pernía-Andrade et al., 2012). Another approach, using deep learning, was introduced by O'Neill et al. (2024). In this approach, a joint convolutional neural network and long short-term memory (CNN-LSTM) network were trained on cerebellar mossy fiber mEPSCs. The output of this model is probabilities as to the existence of synaptic events within a sliding window, which can be thresholded to identify synaptic events. Similarly, Wang et al. (2024) introduce a deep learning approach that trains an artificial neural network (ANN) to differentiate between noise and signals within mEPSC recordings, outputting a confidence value, similar to the CNN-LSTM approach. A peak-finding algorithm is then applied to the confidence values to identify mEPSCs within the recording data. Whilst both deep learning approaches demonstrate promising accuracy in detecting mEPSPs and mEPSCs, they require substantially more data for model training. This makes both approaches less accessible for users wishing to retrain the models to better suit their own training data over the original, potentially unsuitable, training data. Using the Random Forest approach employed by Eventer, we typically train models using a few hundred candidate events from a combination of short segments of multiple representative recordings (note that those short segments are not included in test sets later on). The requirement of Random Forests for less training data in this application may reflect the fact that a small number (currently 10) of carefully chosen features have already been defined to learn the pattern of how these features relate to the user's classification. Distinct from the supervised approaches, Pircher et al. (2022) present an unsupervised approach for detecting spontaneous synaptic currents. This method employs an autoencoder to learn low-dimensional representations of recording segments. These representations are then grouped using K-means clustering to identify common patterns of states in the data. A classifier is then trained to associate the low-dimensional encodings with specific cluster labels to allow the classification of windows to specific states, such as those that contain synaptic events. The advantage of this approach over supervised methods, including Eventer's Random Forest implementation, is that candidates from a training dataset do not need to be expertly labeled. This labeling which can be a time-consuming step, introduces user biases to the models, whether these are intentional or not, as they are in the case of Eventer learning the classification of an expert user. However, the unsupervised approach requires significantly more training data than any of the supervised approaches; training and clustering on such large datasets could be prohibitively computationally expensive. Additionally, the approach could be susceptible to noisy or outlier data, which Random Forest would be more robust to due to its use of ensemble learning.

Although alternative machine learning methods for identifying synaptic events offer certain benefits, Eventer's Random Forest implementation stands out for its streamlined and user-accessible training process and reduced need for annotated data. Additionally, unlike other machine learning approaches, Eventer provides a functioning GUI to aid analysis, along with an approach for semi-automated generation of labeled data during the pre-processing phase by extracting candidate events for users to screen. In fact, Eventer does not necessarily directly compete with recent machine learning approaches. Supervised approaches can still make use of Eventer's candidate event procedure for labeling datasets before being exported for model training. Indeed, such approaches could be incorporated into the framework of Eventer alongside the currently implemented Random Forest to provide a wider array of functionality to users.

Importantly, the Eventer detection methodology could be appropriate for any type of synaptic recording. Indeed, Eventer has been used for the detection of miniature excitatory and inhibitory postsynaptic currents and potentials in brain slices and dissociated neuronal culture within our lab. With relatively small changes to user-accessible analysis settings, users in the lab have also used Eventer to detect synaptic transmission events measured using spontaneous fluorescent synaptic reporters. It is expected that Eventer would also be suitable for use on *in vivo* recordings; however, this has not been tested.

### 4.2 Consistency and reproducibility between users

**Figure 8:**
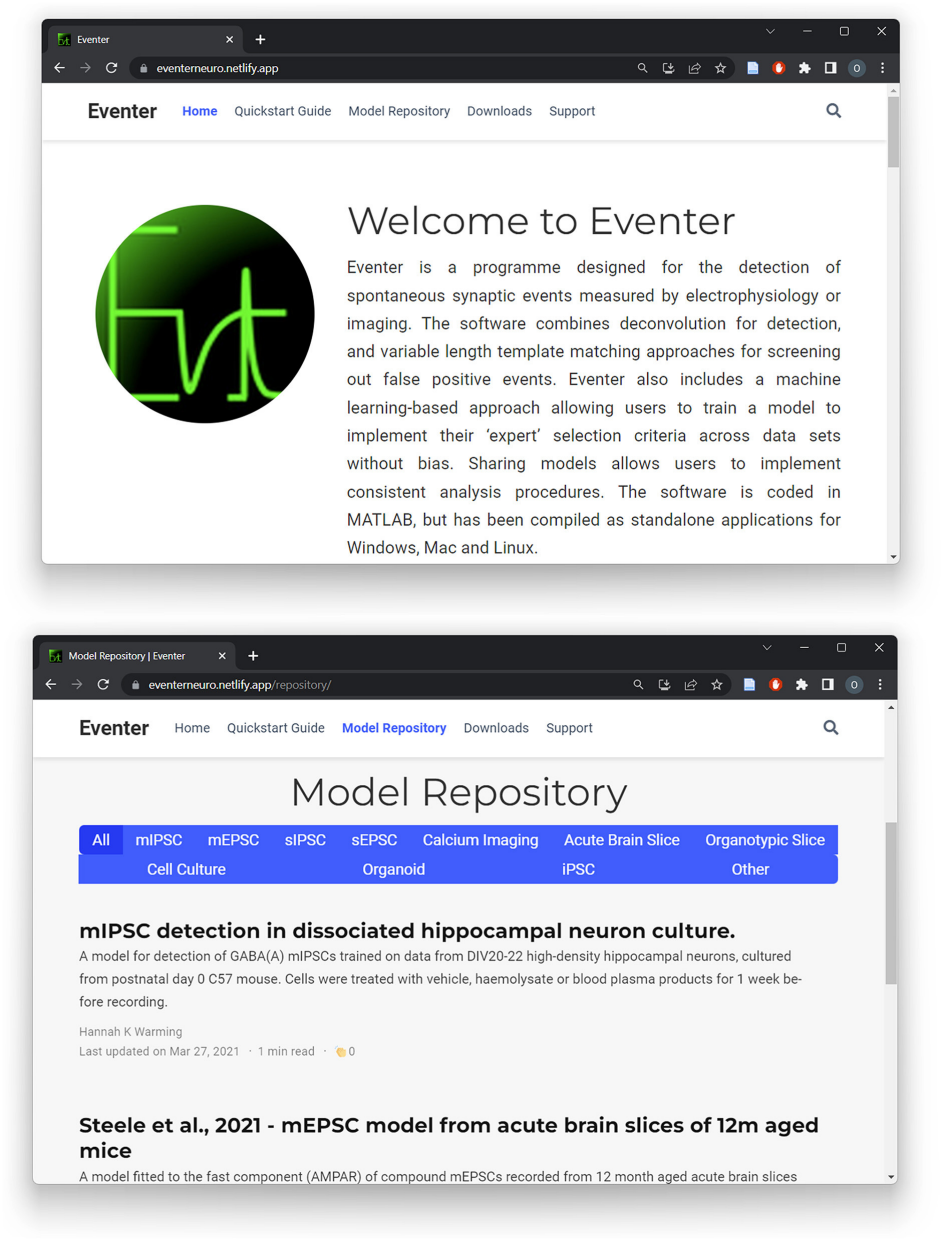
A website for Eventer that includes an online, publicly accessible model repository. A website has been built to increase the accessibility of Eventer, featuring a quick-start guide and full user manual. Additionally, a publicly accessible model repository has been established to enable users to upload and share their own trained models whilst also being able to download the models of others.

Using a single model was shown to increase the consistency between users; however, it is worth noting that the MCC score never reached exactly 1 in any case. This is likely because users were allowed to generate their own initial template from a freely chosen exemplar event from the recording, which slightly changes the candidates available from the initial detection. As such, the model applies the preferences of the expert user to a set of candidate events generated by different sets of event templates. It is indeed possible to save event template settings as a pre-set and pass this onto other users, bundled with a machine learning model if preferable. Nonetheless, these results indicate that machine learning model trained by one expert user can be used by others to replicate that expert user's selection preferences. This therefore inherently increases the reproducibility of analysis between users, which is otherwise a major problem in analyzing electrophysiological data between labs and individuals. To further increase the reproducibility, and indeed accessibility, of analysis between users, a dedicated website for Eventer has been built, as illustrated in Figure 8. This Eventer website (Eventer, 2022) features a quick-start guide, full manual, and download links to the open-source Eventer software. Furthermore, an online model repository has been created to allow users to upload their trained models and for others to download and replicate the selection criteria of other expert users on their own data. A brief description of the conditions the model was trained in is also included here so that those who download the model can use it in appropriate scenarios.

## 5 Concluding remarks

This article describes software for the automated detection and classification of spontaneous synaptic currents, with proof of concept for machine learning-based classification using Random Forests. Training the machine learning models using the implementation of this approach in Eventer can be achieved with relatively little training data and a one-off manual classification step. The software is run from a graphical user interface to facilitate all steps of the analysis, including the training step, and all analysis settings are to facilitate revisiting (and reproducing) specific analyses. Through a series of experiments, we show that Eventer is effective in learning user classification synaptic currents, decreases the time taken to perform analysis, and provides better reproducibility of analysis between users. This software provides a framework that is also capable of integrating additional classification methods using alternative artificial intelligence approaches.

## Data Availability

Eventer software can be downloaded either from the dedicated Sourceforge (https://sourceforge.net/projects/eventer/files/) or GitHub page (https://github.com/acp29/eventer). Data and code relating to the analysis presented in this publication are also available online at GitHub (https://github.com/acp29/Winchester_EVENTER).
